# Influenza A H1 and H3 Transmembrane Domains Interact Differently with Each Other and with Surrounding Membrane Lipids

**DOI:** 10.3390/v12121461

**Published:** 2020-12-17

**Authors:** Szymon Kubiszewski-Jakubiak, Remigiusz Worch

**Affiliations:** Laboratory of Biological Physics, Institute of Physics, Polish Academy of Sciences, 02-668 Warsaw, Poland; kubiszewski@ifpan.edu.pl

**Keywords:** immune response, anti-influenza vaccine, ectodomain orientation, protein–lipid interactions, viral membrane protein

## Abstract

Hemagglutinin (HA) is a class I viral membrane fusion protein, which is the most abundant transmembrane protein on the surface of influenza A virus (IAV) particles. HA plays a crucial role in the recognition of the host cell, fusion of the viral envelope and the host cell membrane, and is the major antigen in the immune response during the infection. Mature HA organizes in homotrimers consisting of a sequentially highly variable globular head and a relatively conserved stalk region. Every HA monomer comprises a hydrophilic ectodomain, a pre-transmembrane domain (pre-TMD), a hydrophobic transmembrane domain (TMD), and a cytoplasmic tail (CT). In recent years the effect of the pre-TMD and TMD on the structure and function of HA has drawn some attention. Using bioinformatic tools we analyzed all available full-length amino acid sequences of HA from 16 subtypes across various host species. We calculated several physico-chemical parameters of HA pre-TMDs and TMDs including accessible surface area (ASA), average hydrophobicity (H_av_), and the hydrophobic moment (µ_H_). Our data suggests that distinct differences in these parameters between the two major phylogenetic groups, represented by H1 and H3 subtypes, could have profound effects on protein–lipid interactions, trimer formation, and the overall HA ectodomain orientation and antigen exposure.

## 1. Introduction

Influenza A virus (IAV) hemagglutinin (HA) remains the key focus of influenza research due to its role in receptor binding, membrane fusion, and formation of the immune response. Due to the lack of proof-reading activity of the IAV RNA-dependent RNA polymerase (RdRP), during genome replication, rapid and small changes occur, which result in a high mutation rate [1]. Progressing accumulation of multiple point mutations in the viral genome is referred to as the antigenic drift, which leads to HA protein plasticity [2]. The emergence of novel antigenic variants is the principal cause of immune escape of IAV and is the reason why influenza vaccinations have to be updated every season. As the key target of the humoral immune response against IAV, HA protein remains the primary component of the influenza vaccine [3].

HA organizes in homotrimers on the surface of the viral membrane [4], which consist of two distinct structural domains, the globular head and the stalk, which roughly correspond to HA1 and HA2 subunits of the mature protein [5]. Being more exposed than the stalk domain, the globular head of HA is the primary target of neutralizing antibodies. It is these head-targeting antibodies, which account for the majority of antibody-mediated immune response. Nevertheless, studies have demonstrated that the less exposed stalk domain can also be targeted by antibodies [6]. Unlike the globular head, which undergoes antigenic drift changes, the more conserved stalk domain evolves at a much slower rate [7,8] and is therefore of interest in the development of the universal influenza vaccine. Based on differences in gene sequence and the resulting antigenic variability, all HAs have been divided into 18 distinct subtypes (H1–H18). Within the subtype, the amino acid sequence similarity of HA is usually estimated to be around 90%, whereas between the subtypes it can fall between 40% and 60% [9]. Phylogenetic analysis of all subtypes revealed that HA proteins can be divided into two major groups: group 1 (H1, H2, H5, H6, H8, H9, H11, H12, H13, H16, H17, and H18) and group 2 (H3, H4, H7, H10, H14, and H15) [9,10,11].

The attachment of the virus, the membrane fusion, as well as the antigenicity of HA so far have been predominantly studied in the context of the ectodomain, protruding from the viral membrane while the HA pre-transmembrane domain (pre-TMD) region (recently described for H1 as the flexible juxtamembrane region or simply the flexible linker [12]) and the transmembrane domain (TMD) region, have been mostly treated as a passive anchor. Studies have shown that HA fusion peptide can interact with the TMD [13,14] although recently contested by [15] and TMD itself is speculated to be involved in the final stages of the fusion of viral and endosomal membranes (reviewed in [16]). Moreover, viral fusion protein TMDs have been shown to increase the membrane lipid order [17] and it has been suggested that TMDs might be associated with lipid rafts [18,19] (cholesterol- and sphingomyelin-enriched membrane platforms), which are crucial for the budding of several envelope viruses. Specifically, HAs of phylogenetic group 2 contain a conserved cholesterol consensus motif (CCM): YKLW [20], which interacts with cholesterol and is essential for virus replication, assembly, and fusion activity [21]. Finally, HA cysteine residues at the TMD/cytoplasmic tail (CL) boundary as well as the CT cysteine residues are predicted to be post-translationally S-acylated and acyl modification of viral fusion proteins has been proposed to be crucial for binding of proteins to membranes and membrane lipid rafts, the final stages of membrane fusion as well as the release of virus particles from infected cells [22,23]. These studies suggest that HA TMD might have an essential function and its interrelationship with the surrounding membrane lipid environment could play an important role in the overall conformation, fusogenicity, and formation of the immune response against the HA.

Of particular interest is the TMD of the H3 subtype. Homologous sequence analysis of TMD regions from 16 HA subtypes has revealed that H3 TMD has two unique cysteine residues (C540 and C544) in its sequence [24]. It has been shown that these cysteine residues might be responsible for various viral characteristics. Mutations in one or both cysteine residues resulted in decreased thermal and acidic stability of the protein, increased growth of the recombinant virus, as well as increased fusion activity [25]. An increase of protein thermal and acidic resistance, a decrease of fusion activity, and an increase in the number of homotrimers were observed, when the native TMD was replaced with that of H3 in a recombinant H9N2 virus [26]. Moreover, replacement of the native TMD with the H3 TMD resulted in enhanced heterosubtypic protection in mice immunized with recombinant HAs (H1, H5, H7, and H9) [27,28,29], recombinant viruses (H7 and H9) [26,28], and virus-like particles (VLPs) (H7) [30]. Interestingly, the TMD substitution did not result in an altered virus assembly or the overall viral protein composition of recombinant viruses in the aforementioned studies. To our knowledge there is no experimental evidence to suggest that the substitution effect on immune response formation is specific to H3 TMD. Taken together, the existing experimental data suggest that the H3 TMD is critical for several viral properties and that the possible correlation between the HA structural conformation and the immune response exists.

The HA TMD substitution can alter the biological characteristics of the recombinant HA and recombinant viruses and thus makes it a very promising tool, not only to study the role of the TMD on the overall structure and function of HA but also to develop a more efficient and possibly universal vaccine against the IAV. However, very little is known about the intrinsic nature of H3 TMD, which could explain its effect. Therefore, in this study, we analyzed several physico-chemical properties of the H3 TMD and pre-TMD and compared them with other HA subtypes at the level of complete proteomes in an attempt to elucidate this phenomenon.

## 2. Materials and Methods

The amino acid sequences of 16 available IAV HA subtypes were downloaded from the OpenFlu database (http://openflu.vital-it.ch/) in FASTA format as separate files according to the host and the subtype (Table S1, Figure S1). Next, they were submitted as the input for the transmembrane region prediction using TMHMM2.0 server (http://www.cbs.dtu.dk/services/TMHMM/). Using custom-written Python v. 3.7 scripts the records of TMHMM-predicted TMDs were created. For further analysis, we applied the procedure of TMD selection performed in other TMD studies [31,32,33]. In short, the TMHMM-predicted TMDs were extended by four amino acids at both ends and the most hydrophobic window of 21 amino acid residues of the TMDs was determined using the GES hydrophobicity scale [34]. The choice of the window size was based on the lengths of TMDs of HA reference strains in UniProt (20 amino acids for both H1 (P26562) and H3 (P03436), respectively). Databases containing all TMDs as well as unique sequences were created as pandas dataframes. The dataset of human single-pass transmembrane proteins was collected in a similar way. FASTA sequences downloaded from UniProt were submitted for TMHMM v. 2.0 prediction and TMDs were selected as in the case of HA sequences. To avoid potential bias, the set was reduced to the sequences having a maximum of 40% identity.

Physico-chemical parameters were calculated using custom-written Python 3.7 scripts (available on request). The accessible surface area (ASA) values for individual amino acids (a set of values for membranous regions of membrane proteins) were taken from previously published work [35]. For the average hydrophobicity (*H_av_*) and the hydrophobic moment (*µ_H_*) calculations, the same hydrophobicity scale (GES) was used. The hydrophobic moment was calculated as:

$$\mu_{H}={\left\{{\left(\overset{n}{\underset{k=1}{\sum }}H_{k}\sin\left(k\delta \right)\right)}^{2}+{\left(\overset{n}{\underset{k=1}{\sum }}H_{k}\cos\left(k\delta \right)\right)}^{2}\right\}}^{1/2}\cdot \frac{1}{n}$$

The *µ_H_* for TMDs were calculated for the entire lengths (*n* = 21) with the turn angle *δ* = 100° corresponding to the ideal α-helical structure. In the case of the hydrophobic moment maps the *µ_H_* was calculated for δ in range from 0° to 180° with the step of 10° for the window of *n* = 7 residues. The *µ_H_* value in the map corresponds to the middle of the seven-residue window. To characterize the differences between the subtypes, we focused on the parameters calculated for individual sequences. However, the same conclusions could be drawn using the whole sets of HA TMDs with reduced identity to 40% (Figure S2). Sequence alignments were performed using the algorithm implemented at the EMBOSS Needle server (https://www.ebi.ac.uk/Tools/psa/emboss_needle/) with default parameters [36]. Seaborn package (https://seaborn.pydata.org/index.html) was used for plotting, Chimera (http://www.cgl.ucsf.edu/chimera) for structural rendering, and Inkscape for figure assembly.

## 3. Results

### 3.1. HA TMDs Differ between H1 and H3 Subtypes

We assembled a comprehensive set of TMD sequences of the HA protein according to the subtype and the host by separating the records into three categories: human, other mammalian, and avian. The total numbers of records in the OpenFlu database reflected the occurrence of viral strains circulating among hosts: for humans and other mammals the maximum number of TMD records was observed for H1 and H3 subtypes (Figure S1), whereas avian HA populated mainly the H9, H5, and H3 subtypes. Noticeably, H3 subtype was populated by all three host categories.

Next, to avoid the possible overabundance of certain close homologs, we performed further analysis for the TMD sequences, which appear the most frequently in each subtype (Table 1 and Table S1, Figure S1). For a given subtype the sequences were almost the same in all hosts analyzed. The only difference was observed in human host: L15 to V15 substitution in H1 and V15 to A15 substitution in H3 (numbers refer to the position within the TMD). However, for all hosts the H3 TMD differs quite strikingly from the H1 TMD by e.g., the presence of tryptophan, two cysteines, and higher leucine/valine (*L/V*) ratio. Pairwise sequence alignment of the TMD sequences for H1 and H3 subtype (Table 1) resulted in 45.5% similarity and 38.1% identity for human host HA and 50% similarity and 31.8% identity for the other mammalian and avian hosts.

### 3.2. Physico-Chemical Parameters of HA TMDs Differ between Phylogenetic Groups

**Figure 1:**
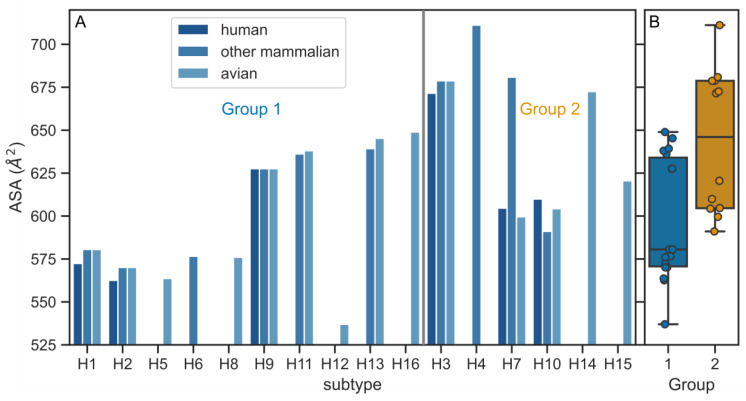
(**A**) Accessible surface area (ASA) values for individual hemagglutinin (HA) TMD sequences according to the subtype and host. (**B**) Average ASA values for the subtypes belonging to the two phylogenetic groups. The difference between groups 1 and 2 is statistically significant (*p* < 0.01, two-sided Mann–Whitney U test).

To see whether the differences between the H1 and H3 TMDs are reflected in their physico-chemical properties we performed further analysis for extended set of subtypes. First, we calculated the accessible surface area (ASA) for individual TMD records according to the host and phylogenetic group (Figure 1). Since our dataset consisted of TMDs of equal lengths, a direct comparison of ASA values was possible. The averaged ASA value (for all three host classes) for the H1 subtype was 577.9 ± 4.7 Å^2^ which is substantially smaller than that for the H3 subtype (676.3 ± 4.2 Å^2^). This observation held for the averaged values of the HA TMD belonging to the two phylogenetic groups with the average values of 599 ± 36 Å^2^ and 544 ± 42 Å^2^ for groups 1 and 2, respectively (Figure 1).

**Figure 2:**
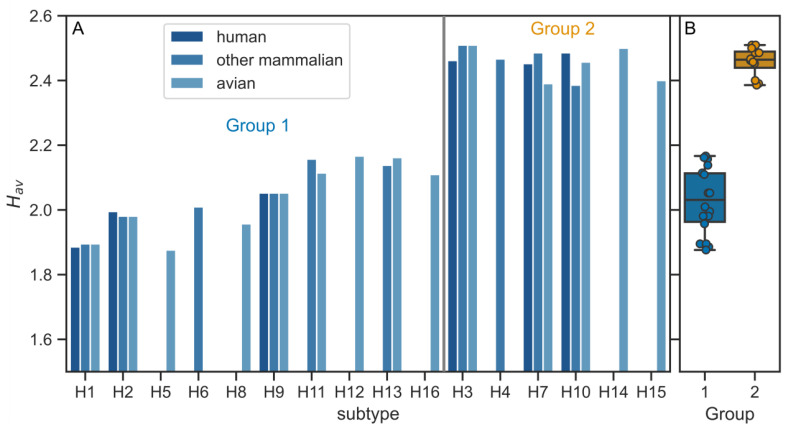
(**A**) Average hydrophobicity (H_av_) values for individual HA TMD sequences according to the subtype and host. (**B**) Average H_av_ values for the subtypes belonging to the two phylogenetic groups. The difference between groups 1 and 2 is statistically significant (*p* < 10^−5^, two-sided Mann–Whitney U test).

The TMD sequences in H1 and H3 subtypes differed in the number of polar amino acids, which had an impact on the overall hydrophobicity. The H1 TMD contained five polar residues (four serines, one threonine), whereas in H3 TMD this number was reduced to 2 (two serines). Intrigued by these compositional differences, we performed further calculations of the TMD average hydrophobicity (H_av_) (Figure 2). Interestingly, the highest H_av_ values (i.e., having the largest hydrophobicity) appeared for the subtypes which belong to the phylogenetic group 2 with minor differences between host types (Figure 2). The averaged values (GES scale) were 2.03 ± 0.10 and 2.46 ± 0.04 for groups 1 and 2, respectively.

**Figure 3:**
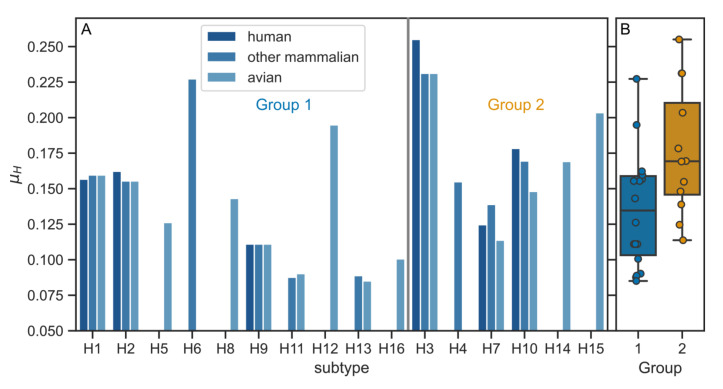
(**A**) Hydrophobic moment (µ_H_) values for individual HA TMD sequences according to the subtype and host. (**B**) Average μ_H_ values for the subtypes belonging to the two phylogenetic groups. The difference between groups 1 and 2 is statistically significant (*p* < 0.01, two-sided Mann–Whitney U test).

Since it is known that single-pass transmembrane domains adopt a helical conformation, we checked whether the different positioning of polar residues observed in H1 and H3 TMDs affects the helix amphiphilicity. As its measure, we calculated a commonly used hydrophobic moment (µ_H_) with the turn angle of 100° corresponding to an ideal α-helix structure [33,37]. It is defined in the way that the more amphipathic the helix is, the larger the µ_H_. Indeed, the average amphiphilicity of the group 2 TMDs was larger as compared to the group 1 (0.18 ± 0.05 and 0.13 ± 0.04, respectively) (Figure 3). Noticeably, the µ_H_ value was the largest for H3 TMD (0.239 ± 0.014, averaged for the three host classes), which is significantly larger than the corresponding value for H1 (0.159 ± 0.002).

### 3.3. Comparison with the Human Single-Pass TMDs

**Figure 4:**
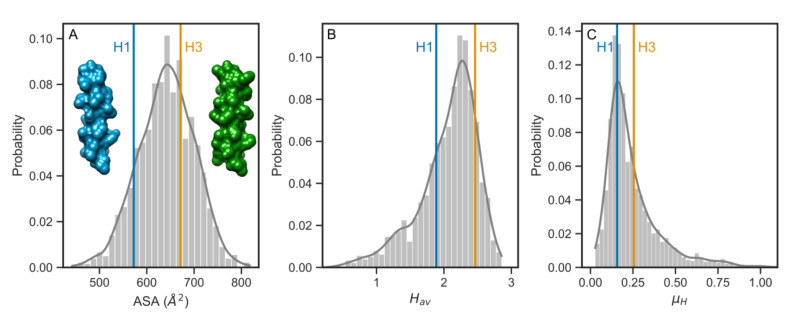
The distribution of (**A**) ASA, (**B**) H_av_, and (**C**) µ_H_ values for human single-pass TMDs with the corresponding values for human H1 and H3 TMDs. (**A**) Surface representations of H1 and H3 TMDs (α-helices built based on sequences).

Having observed that all three physico-chemical parameters had larger average values for the phylogenetic group 2 subtypes, we decided to look closer at the differences between human H1 and H3 subtypes in the context of single-pass transmembrane domains of human proteins. Currently, predominant circulating IAVs are A/H1N1 and A/H3N2 and it has been recommended by the WHO that these viruses are included in the formulation of the seasonal vaccine [38]. Therefore, we collected a dataset of single-pass TMD of the same length as for viral HA and reduced it to the level of 40% identity to avoid potential sequential bias. Figure 4 shows histograms of ASA, H_av_, and µ_H_ parameters with the values for human H1 and H3 TMDs. The histogram for ASA had a symmetric shape (Figure 4A), whereas the histograms for H_av_ and µ_H_ had more long-tailed shapes for lower H_av_ and higher µ_H_ values, respectively (Figure 4B,C). In all three cases, however, the position of values for H1 TMD were located on the left-hand side of the distribution of parameters, in contrast to the corresponding higher values for H3 TMD on the right.

### 3.4. The Pre-TMD Region Also Differs between H1 and H3 Subtypes

**Figure 5:**
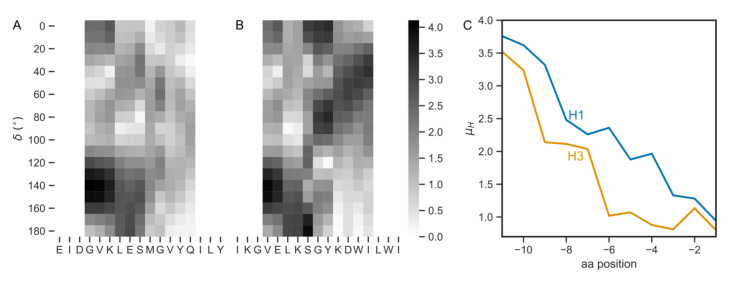
The µ_H_ maps for the pre-transmembrane (pre-TMD) fragments in (**A**) H1 and (**B**) H3 human subtypes. The squares in grayscale depict the µ_H_ values for a seven amino acid fragment centered for the middle residue. (**C**) The cross sections of maps for δ = 140°.

Recent structural studies have shown that the TMD region of H1 HA is linked to the ectodomain by a juxtamembrane flexible linker region or pre-TMD. This fragment appeared to be involved in tilting the H1 HA ectodomain with respect to the membrane surface (demonstrated in the detergent micelle as well as lipid bilayer) [12]. To see whether sequence variations are present likewise in pre-TMDs, we performed the analysis of those regions. First, we collected the sequences of 11 amino acids upstream of the TMD regions (Table S3 contains the most frequently occurring sequences). The sequences corresponding to human H1 and H3 subtypes (GVKLESTRIYQ and VELKSGYKDWI, respectively) are identical in 28.6% and similar in 50%. To assess whether the pre-TMDs differ in amphiphilicity, we performed calculations of µ_H_. Since the structure of the pre-TMD of H1 HA is not α-helical, we extended the calculations to a range of turn angles (δ) from 0° to 180°. Such approach allows for finding the turn angle values resulting in maximal µ_H_ values, often corresponding to the secondary structure elements [33,39,40]. Figure 5A,B shows the hydrophobic moment maps for human H1 and H3 pre-TMDs, respectively. For both subtypes the maximum µ_H_ values were observed for δ = 140°. Interestingly, the averaged δ value calculated from φ and ψ dihedral angles (PDB: 6HJR) for the *N*-terminal Asp174-Leu178 fragment gave a close value (130°). Next, to compare µ_H_ values between H1 and H3 subtypes, we plotted the hydrophobic moment map cross sections for δ = 140° (Figure 5C). It can be seen that µ_H_ values for H1 subtype were larger than for H3, meaning that the H1 pre-TMD was more amphipathic.

## 4. Discussion

The findings in this study are the first attempts to link the physico-chemical properties of pre-TMDs and TMDs of IAV HAs, belonging to phylogenetic group 1 (H1) and group 2 (H3), with their behavior in the membrane bilayer. Plausibly distinct oligomerization properties, different interactions with the surrounding lipids, and varying physico-chemical properties of these HA elements may influence the overall HA conformation and antigen exposure.

Our average hydrophobicity (H_av_) and hydrophobic moment (µ_H_) analysis pointed out that H3 TMD is more hydrophobic and more amphipathic than H1 TMD (with the same observations for average values for entire phylogenetic groups). From the existing structure of H1, it is known that some of the polar residues are facing the inner site of the H1 TMD trimer in a hydrophobic membrane milieu (Figure S2). Although H1 TMD is more rich in serines as compared to H3 TMD, they are not organized in any of the known serine-including dimerization motifs like SxxSSxxT or SxxxSSxxT [41]. However, for the more hydrophobic H3 TMD, the possible noncovalent bonds established between polar residues might have even larger favorable enthalpic contribution to the overall trimer stability as compared to H1 trimer. We rationalize this argument by former studies of TMD dimers in which erythropoietin receptor (EpoR) dimers were observed in the membranes of giant plasma membrane vesicles, while the homo dimers of interleukin-4 receptor chains (IL-4Rα and IL-2Rγ, respectively) were not detected [42]. In the 21 amino acid hydrophobic stretch of TMD fragments all three receptors contained four polar amino acids, however, the EpoR was the most hydrophobic. Thus, the polar residues in more hydrophobic H3 TMD (as compared to H1 TMD) may play a similar role by creating more energetic noncovalent bonds.

Distinct oligomerization properties of the TMDs appear also at the level of primary structure (reviewed in [43]). Some of the aromatic residues, which are more numerous in H3 TMD, are organized in FxxS motive, shown to promote transmembrane helix dimerization [44]. The same phenylalanine is a part of a preceding ‘aromatic-xx-aromatic’ sequence (WxxF), also promoting intrahelical interactions [45]. Indeed, phenylalanine rings were proposed to be involved in intrahelical stacking interactions in the model of H3 TMD trimer [46]. Other aromatic residues-containing motifs, such as WAA, YAA, FAA [45] are not present in the TMD sequence of H3 subtype. Whether the aromatic or polar residues are indeed involved in the H3 TMD trimer stabilization will be revealed by future structural studies.

Our observations regarding TMD accessible surface area (ASA), which appeared to be larger in H3 subtype as compared to H1, may reflect different affinity to membrane rafts. Recently, TMD ASA was described as a determinant of raft affinity, namely the TMDs with smaller ASA were found to be preferentially located in a raft-like environment (mimicked by liquid-ordered (L_o_) domain of giant plasma membrane vesicles) [47]. Application of that relationship would imply that the H3 TMD has weaker raft association than H1 TMD and would accommodate more easily in more disordered membranes. Interestingly, more unsaturated lipid chains were observed in avian brain and kidney tissues as compared to mammalian tissues [48,49], however, further studies are required to verify whether more bulky H3 TMD is related to a wider host range of the H3 IAV. On the other hand, higher ASA values of the H3 TMD may be compensated by the conserved cholesterol consensus motif (CCM) YKLW. The CCM might be regarded as one of the factors responsible for the protein location in membrane rafts which are cholesterol- and sphingomyelin-enriched (recently reviewed in [50]). Indeed, mutations of the CCM resulted in decreased H3 IAV replication, membrane fusion, and affected virus assembly [21]. However, raft nanodomain location was reported for HAs representing both phylogenetic groups: H2 (group 1) [18] and H3 (group 2) [19] and membrane rafts were indicated as a platform for virus assembly and budding [51,52,53].

Apart from protein–lipid interactions, membrane raft affinity can be driven by acylation (i.e., palmitoylation or stearation), as implicated by a number of previous studies [50]. Indeed, acylation of HA has been shown to be important for virus growth [54,55] and directly evidenced to be involved in virus release for H3 subtype [56]. The cytoplasmic cysteines of HA are palmitoylated, whereas stearate is exclusively attached to the cysteine positioned in the TMD/CT boundary of HA ([57], reviewed in [23]). The presence of two cysteines in the H3 TMD, which can be potential acylation sites, possibly facilitates raft association, again compensating larger ASA values. However, it is important to emphasize that no experimental data exists to confirm that H3 TMD cysteines are acylated. Nevertheless, such dual membrane anchors might be involved in suppositional border location of membrane rafts, suggested recently for single-pass transmembrane adaptor protein linker for activation of T cells (LAT) [58]. Direct protein–lipid interactions have been recently pointed as responsible for selective incorporation of proteins into HIV envelope [59]. It is conceivable that the putative border location of HA TMD would contribute to the increased line tension of the membrane clusters inducing lipid acyl chains deformations and lowering the energy barrier for membrane fusion or fission. Other contributions to more efficient fusiogenicity of H3 TMD might lie in higher L/V ratio (as compared to H1 TMD), since peptides with such features were shown to have increased fusion activity [60].

**Figure 6:**
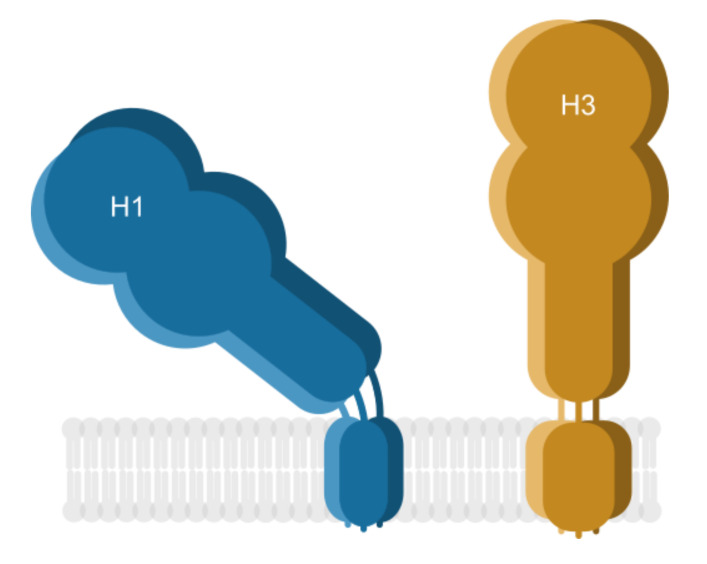
Summary: position of the ectodomain in respect to membrane. Cartoon representing H1 was drawn based on the existing structure (PDB ID: 6HJR); the conformation of H3 is hypothetic.

In recent years the remarkable diversity of eukaryotic lipids is drawing an increasing research interest. Lipid membrane is no longer regarded as a passive environment serving as an anchor for proteins; in contrast, there is more and more evidence of lipid influence on protein structure and function (reviewed in [61]). However, the underlying mechanisms remain largely unexplored. One of the best studied examples hitherto is Ire1 protein responsible for the unfolded protein response (UPR) [62]. It uses a juxtamembrane, amphipathic helix to sense membrane aberrancies and control UPR activity. Based on the described mechanism of Ire1 action, we hypothesize that the interactions of TMDs and pre-TMDs of HA lead to different positions of the H1 and H3 ectodomains with the following rationale behind. Lower µ_H_ of the H3 pre-TMD is possibly responsible for in-line positioning of the pre-TMD with the TMD axis, whereas larger µ_H_ (more amphipathic) H1 pre-TMD is more susceptible to adopt a tilted conformation as it is seen in the structural studies [12]. Similar conformations were observed for the two point mutations of Ire1 juxtamembrane linker (F531R and V535R), which substantially change the amphipathic character of that protein fragment. We calculated the hydrophobic moment maps for the wild-type Ire1 and the two mutants (Figure S4): wild-type, ‘straight’ protein fragment had lower µ_H_ values, whereas the two mutants exhibiting tilted conformations resulted in larger µ_H_ values. In this case no particular turn angles giving maxima were observed, however large conformational plasticity was observed in the amphipathic region of V535R (Video S2 [62]) in contrast to the wild-type (Video S1 [62]). Thus, we speculate that the H3 pre-TMD could potentially position the H3 ectodomain perpendicularly in relation to the viral membrane, in contrast to the observed, tilted conformation of H1 (Figure 6).

It is becoming more evident that the protein stability of the antigen such as HA can greatly influence its immunogenicity. Studies have shown that fusion proteins consisting of the HA ectodomain and a stabilizing region such as bacterial T4 phage fibritin foldon domain [63], GCN4pII trimerization repeat [64], or ferritin [65] resulted in an increase in HA stability, which in turn improved the cross-reactive immunity. In a different study, the HA TMD was replaced with glycosyl-phosphatidylinositol (GPI), which decreased the fusion activity of the protein and impaired its structure and ability to form homotrimers [66,67]. Two conserved and unique cysteine residues (C540 and C544), found in the H3 TMD, could explain its inherent stability via formation of a intermolecular disulfide bond [68]. Several other studies from the Cao group also link the stability of the H3 protein to its broadened immunogenic effect. However, the underlying cause of this effect is still unknown. We hypothesize that the interactions of TMDs and pre-TMDs lead to different positions of the H1 and H3 ectodomains. This could allow for the conserved stalk region epitopes of the H3 ectodomain to be more exposed and be easily accessible during the recognition by B lymphocytes. Such breaking of the HA globular head immunodominancy could account for the increased humoral immune response to H3 TMD-containing recombinant proteins and viruses and the heterosubtypic protection they have been observed to confer. This model, however, remains hypothetical and needs to be tested experimentally.

## Figures and Tables

**Table 1:** Transmembrane domain (TMD) sequences for H1 and H3 subtypes. The occurrence is calculated as the number of a given sequence in the OpenFlu dataset divided by the total number of sequences for a given subtype and host. Amino acids in bold indicate identical amino acids in the aligned sequences.

Subtype	Host	Occurrence (%)	Sequence
H1	Human	63.1	ILAIYSTVASSLVLVVSLGAI
H1	Other mammalian	38.9	ILAIYSTVASSLVLLVSLGAI
H1	Avian	85.6	ILAIYSTVASSLVLLVSLGAI
H3	Human	67.1	-LWISFAISCFLLCVALLGFIM
H3	Other mammalian	55.0	-LWISFAISCFLLCVVLLGFIM
H3	Avian	79.4	-LWISFAISCFLLCVVLLGFIM

## Supplementary Materials

The following are available online at https://www.mdpi.com/1999-4915/12/12/1461/s1, Figure S1. Numeric characteristics of the TMDs analyzed. Figure S2: Structure of the H1 TMD trimer. Figure S3: Distributions of physico-chemical properties calculated for complete, homology-reduced TMDs. Figure S4. Hydrophobic moment maps for the pre-TMD region of the (A) wild-type Ire1 protein [61] and F531R and V535R mutants, (B) and (C) respectively. Table S1: Numbers of the TMD segments according to the host and subtype, Table S2: TMD sequences with the highest frequency of occurrence according to the host and subtype. Table S3: Pre-TMD sequences with the highest frequency of occurrence according to the host and subtype.
