# Supplementary material for: Influenza A H1 and H3 Transmembrane Domains Interact Differently with Each Other and with Surrounding Membrane Lipids
Source: Viruses. 2020 Dec 17;12(12):1461. doi: 10.3390/v12121461 (PMC7765950; doi:10.3390/v12121461)
Supplement: Supplementary file 1 [file viruses-12-01461-s001.pdf]

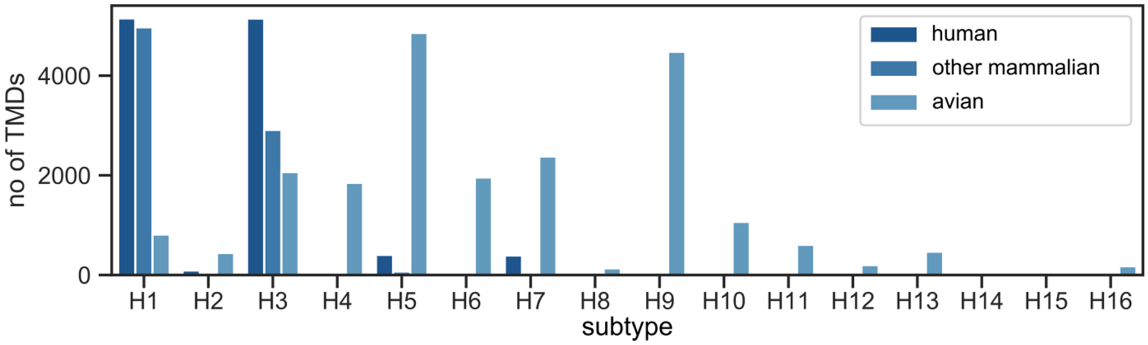

Figure S1. Numeric characteristics of the TMDs analyzed. Bar plots show the total number of TMD records from the OpenFlu database according to the subtype and host.

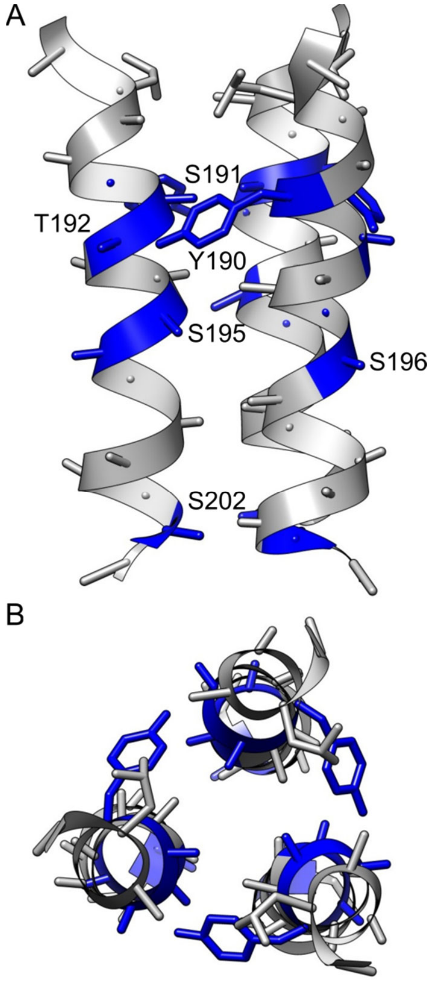

Figure S2. Structure of the H1 TMD trimer. A) side and B) top views. Polar residues are shown in blue (numbering according to PDB ID: 6HJQ). Side chain atoms are not completely resolved. The structure of the H3 TMD trimer is not available.

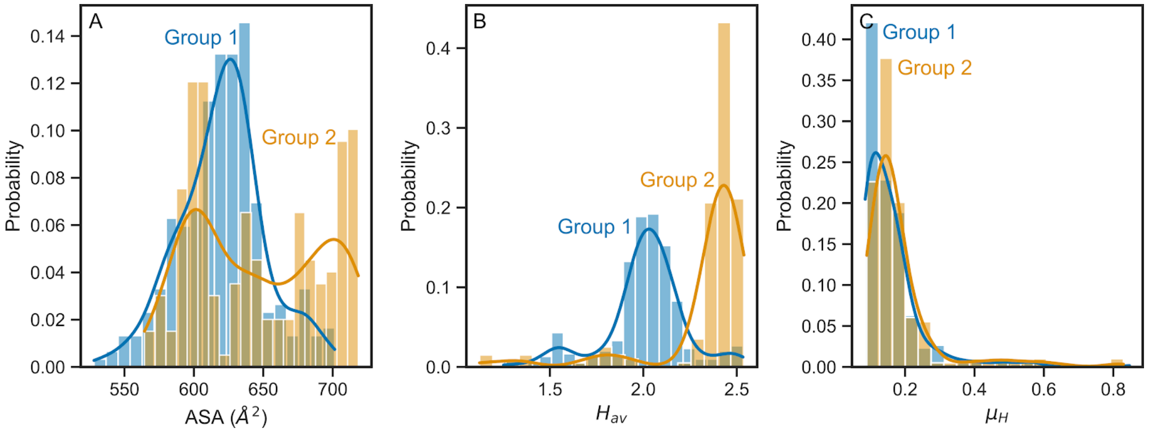

Figure S3. Distributions of A) ASA, B) H_{av} and C) μ_H values calculated for the dataset of TMDs (all hosts combined) with homology purged to 40% sequence identity. The differences between two phylogenetic groups are statistically significant ($p < 0.001$, two sided Mann-Whitney U test).

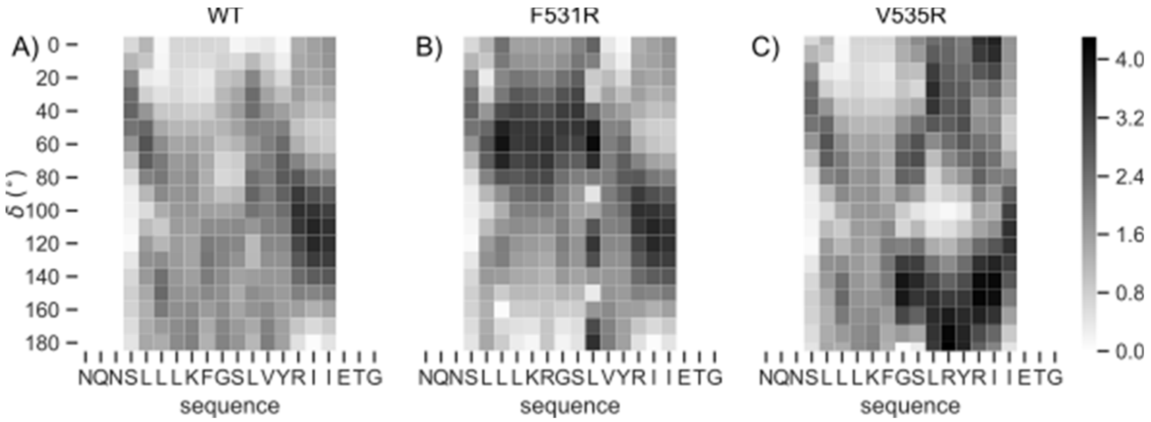

Figure S4. Hydrophobic moment maps for the pre-TMD region of the A) wild-type Ire1 protein [61] and F531R and V535R mutants, B) and C) respectively. In molecular dynamics simulations, both mutants show a tilted pre-TMD regions with respect to the TMD helix, in contrast to the wild-type protein.

Table S1. Total number of TMD sequences.

Serotype	Host	No of TMDs
H1	human	5148
H1	other mammalian	4969
H1	avian	812
H2	human	91
H2	other mammalian	3
H2	avian	442
H3	human	5145
H3	other mammalian	2911
H3	avian	2066
H4	human	0
H4	other mammalian	9
H4	avian	1849
H5	human	403
H5	other mammalian	70
H5	avian	4857
H6	human	0
H6	other mammalian	3
H6	avian	1957
H7	human	392
H7	other mammalian	22
H7	avian	2378

H8	human	0
H8	other mammalian	0
H8	avian	134
H9	human	24
H9	other mammalian	36
H9	avian	4477
H10	human	6
H10	other mammalian	2
H10	avian	1066
H11	human	0
H11	other mammalian	1
H11	avian	606
H12	human	0
H12	other mammalian	0
H12	avian	199
H13	human	0
H13	other mammalian	2
H13	avian	468
H14	human	0
H14	other mammalian	0
H14	avian	28
H15	human	0
H15	other mammalian	0
H15	avian	18
H16	human	0
H16	other mammalian	0
H16	avian	177

Table S2. TMD sequences with the highest frequency of occurrence according to the host and subtype.

Serotype	Host	Occurrence (%)	Sequence
H1	human	63.1	ILAIYSTVASSLVLVVSLGAI
H1	human	34.9	ILAIYSTVASSLVLLVSLGAI
H1	human	0.3	MGVYQILAIYSTVASSLVLLV
H1	other mammalian	38.9	ILAIYSTVASSLVLLVSLGAI
H1	other mammalian	36.3	ILAIYSTVASSLVLVVSLGAI
H1	other mammalian	3.7	ILSIYSTVASSLVLLVSLGAI
H1	avian	85.6	ILAIYSTVASSLVLLVSLGAI
H1	avian	6.2	ILAIYSTVASSLIWLVSLGAI
H1	avian	1.8	ILAIYSTVASSLVLLVSLGAL
H2	human	74.7	ILAIYATVAGSLSLAIMMAGI
H2	human	15.4	ILAIYATVAGSLSLAIMIAGI
H2	human	8.8	ILAIYATVAGSLSLAIMVAGI
H2	other mammalian	100.0	ILAIYATVAGSLSLAIMIAGI
H2	avian	90.0	ILAIYATVAGSLSLAIMIAGI
H2	avian	5.2	ILAIYATVAGSLSLAIMMAGI
H2	avian	1.4	YATVAGSLSLAIMIAGIFLWM
H3	human	67.1	LWISFAISCFLLCVALLGFIM
H3	human	23.5	LWISFAISCFLLCVVLLGFIM
H3	human	4.9	LWISFAISCFLLCIVLLGFIM
H3	other mammalian	55.0	LWISFAISCFLLCVVLLGFIM
H3	other mammalian	9.6	LWISFAISCFLICVVLLGFIM
H3	other mammalian	7.8	LWISFAISCFLLCVALLGFIM
H3	avian	79.4	LWISFAISCFLLCVVLLGFIM
H3	avian	8.7	LWISFAISCFLLCVVILGFIM
H3	avian	3.3	ILWISFAISCFLLCVVLLGFI
H4	other mammalian	66.7	ILWISFSISCFLLVALLLAFI
H4	other mammalian	22.2	ILWFSFSISCFLLVALLLAFI
H4	other mammalian	11.1	ILWISFSISCFLLVALLLAFV
H5	avian	43.2	ILSIYSTVASSLALAIMVAGL
H5	avian	12.5	ILSIYSTVASSLALAIMIAGL
H5	avian	11.7	ILSIYSTVASSLVLAIMMAGL
H6	other mammalian	66.7	ILAIYSTVSSSLVLVGLIMAM
H6	other mammalian	33.3	ILAIYSTVSSSLVLVGLILAM
H7	human	95.4	FSFGASCFILLAIVMGLVFIC
H7	human	1.8	FSFGASCFILLAIAMGLVFIC
H7	human	1.3	FSFGASCFLLLAIAMGLVFIC
H7	other mammalian	50.0	IILWFSFGASCFLFLAIAMVL
H7	other mammalian	18.2	FSFGASCFLLLAIAMGLVFIC
H7	other mammalian	9.1	FSFGASCFLFLAIAMGLVFIC
H7	avian	35.5	FSFGASCFLLLAIAMGLVFIC
H7	avian	28.6	FSFGASCFILLAIAMGLVFIC
H7	avian	23.8	FSFGASCFILLAIVMGLVFIC
H8	avian	95.5	IYSTVAASLCLAILIAGGLIL
H8	avian	1.5	IYSTVAASLCLAILVAGGLIL
H8	avian	0.7	STVAASLCLAILIAGGLILGM
H9	human	62.5	IYSTVASSLVIAMGFAAFLFW
H9	human	29.2	IYSTVASSLVLAMGFAAFLFW
H9	human	4.2	STVASSLMLAIGFAAFMFWVM
H9	other mammalian	80.6	IYSTVASSLVIAMGFAAFLFW
H9	other mammalian	11.1	IYSTVASSLVVAMGFAAFLFW
H9	other mammalian	5.6	IYSTVASSLVLAMGFAAFLFW
H9	avian	73.2	IYSTVASSLVIAMGFAAFLFW
H9	avian	12.0	IYSTVASSLVLAMGFAAFLFW
H9	avian	1.5	IYSTVASSLVVAMGFAAFLFW
H10	human	100.0	FSFGASCFVLLAVVMGLFFFC
H10	other mammalian	50.0	FSFGASCFVLLAAVMGLVFFC
H10	other mammalian	50.0	FSFGASCFVLLAVVMGLVFFC
H10	avian	56.1	FSFGASCFVLLAVIMGLVFFC
H10	avian	34.4	FSFGASCFVLLAVVMGLVFFC

H10	avian	1.5	FSFGASCFVLLAVVMGLFFFC
H11	other mammalian	100.0	IYSCIASSLVLAALIMGFMFW
H11	avian	36.0	IYSCIASSLVLAAIIMGFILW
H11	avian	29.9	IYSCIASSLVLAAIIMGFIFW
H11	avian	26.9	IYSCIASSLVLAALIMGFILW
H12	avian	88.4	SSVASSLVLLLMIIGGFIFGC
H12	avian	11.6	IYSSVASSLVLLLMIIGGFIF
H13	other mammalian	100.0	IYSCIASSVVLVGLILAFIMW
H13	avian	66.5	IYSCIASSIVLVGLILAFIMW
H13	avian	15.0	IYSCIASSVVLVGLILSFIMW
H13	avian	11.8	IYSCIASSVVLVGLILAFIMW
H14	avian	71.4	IILWISFSMSCFVFVALILGF
H14	avian	21.4	ILWISFSMSCFVFVALILGFV
H14	avian	3.6	LWISFSMSCFVFVALILGFVF
H15	avian	44.4	WFSFGASCVMLLAIAMGLIFM
H15	avian	44.4	WFSFGASCFMLLAIAMGLIFM
H15	avian	11.1	WFSFGASCFVLLAIAMGLIFM
H16	avian	36.7	IYSCIASSVVLVGLILAFILW
H16	avian	29.9	IYSCIASSTVLVGLILAFIMW
H16	avian	9.0	IYSCIASSIVLVGLILAFIMW

Table S3. Pre-TMD sequences with the highest frequency of occurrence according to the host and subtype.

Serotype	Host	Occurrence (%)	Sequence
H1	human	63.3	ESTRIYQ
H1	avian	91.5	ESMGVYQ
H1	other mammalian	43.7	ESTRIYQ
H2	human	91.2	SSMGVYQ
H2	avian	86.2	SNMGVYQ
H2	other mammalian	100.0	SNMGVYQ
H3	human	95.9	SGYKDWI
H3	avian	91.5	SGYKDWI
H3	other mammalian	44.7	SGYKDWI
H4	avian	91.8	TQGYKDI
H4	other mammalian	66.7	TQGYKDI
H5	human	43.9	ESIGTYQ
H5	avian	42.7	ESIGTYQ
H5	other mammalian	57.1	ESIGTYQ
H6	avian	52.6	ENLGVYQ
H6	other mammalian	33.3	ENLGVYQ
H7	human	96.9	YKDVILW
H7	avian	50.4	YKDVILW
H7	other mammalian	54.5	LSSGYKD
H8	avian	94.8	TTYKILS
H9	human	91.7	GTYKILT
H9	avian	85.8	GTYKILT
H9	other mammalian	75.0	GTYKILT
H10	human	100.0	YKDIILW
H10	avian	58.0	YKDVILW
H10	other mammalian	100.0	YKDIILW
H11	avian	49.2	NVYKILS
H11	other mammalian	100.0	NVYKILS
H12	avian	88.4	YKILSIY
H13	avian	40.5	NVYKALS
H13	other mammalian	100.0	NVYKALS
H14	avian	46.4	LTMGYKD
H15	avian	50.0	GYKDVIL
H16	avian	82.5	NVYKILS
